# 임신성 당뇨병을 가진 임산부를 위한 간호중재 프로그램의 효과: 무작위 대조군 실험연구의 체계적 문헌고찰

**DOI:** 10.4069/kjwhn.2021.03.02

**Published:** 2021-03-20

**Authors:** JooHee Kim, ChaeWeon Chung

**Affiliations:** 1 Seoul St. Mary’s Hospital, College of Medicine, The Catholic University of Korea, Seoul, Korea; 1 서울성모병원; 2 Research Institute of Nursing Science, College of Nursing, Seoul National University, Seoul, Korea; 2 서울대학교 간호대학 간호과학연구소

**Keywords:** Gestational diabetes, Nursing, Program, Randomized controlled trial, Systematic review, 임신성 당뇨병, 간호 중재, 무작위 실험 연구, 체계적 문헌고찰

## Abstract

**Purpose:**

This study aimed to identify the effects of nursing intervention programs for women with gestational diabetes mellitus (GDM) through a critical review of recent studies.

**Methods:**

Studies related to effects of nursing intervention programs for women with GDM published in English or Korean between 2000 and 2019 were extracted from 10 electronic databases. The quality of the studies was evaluated and double-checked for accuracy by two reviewers using the Revised Cochrane Risk-of-Bias tool for randomized controlled trials.

**Results:**

Twenty studies were selected, of which 19 had a low risk of bias and one had a high risk of bias. Interventions fell into six main groups: (1) integrated interventions, (2) self-monitoring of blood glucose levels, (3) dietary interventions, (4) exercise, (5) psychotherapy, and (6) complementary therapy. This review found that nursing interventions for GDM were of many types, and integrated interventions were the most common. However, low-carbohydrate diets and blood glucose monitoring interventions did not show statistically significant results. Evidence shows that various nursing intervention programs applied to GDM improved diverse aspects of maternal, fetal, and neonatal health, including both physical and psychological aspects.

**Conclusion:**

The composition and delivery of integrated interventions continue to evolve, and these interventions affect physical and psychological indicators. Although interventions affecting physical health indicators (e.g., blood glucose levels, diet, and exercise) are important, many studies have shown that programs including psycho-emotional nursing interventions related to anxiety, depression, stress, self- efficacy, and self-management are also highly useful.

## Introduction

### 연구 필요성

임신성 당뇨병은 임신 기간에 처음으로 발생하거나 진단되는 포도당 과민증의 모든 단계를 말한다[1]. 국내 보건의료 빅데이터 개방시스템에 따르면 임신성 당뇨병을 진단받은 임산부가 2010년 37,072명에서 2019년 52,752명으로 약 1.4배 증가하였고, 이 중 35세 이상의 임산부는 8,948명(2010년)에서 20,938명(2019년)으로 2배 늘어난 것으로 나타났다. 한편 임신성 당뇨병의 위험요인은 고령, 비만, 가족력, 이전 임신성 당뇨병 또는 당뇨병 이력, 다낭성 난소 증후군 등으로 알려져 있는데[2], 이 중 특히 우리나라 임산부의 임신성 당뇨병 고위험요인으로 지목되는 문제는 비만과 고령 임신이다. 국내의 평균 출산 연령이 33세이고, 35세 이상 임산부의 비중이 33.4%로 10년 전에 비해 2.2배 증가한 것[3]과 19세 이상 여성의 비만율이 2018년에 25.5%로 지속해서 높게 유지되는 추이를 볼 때[4], 우리나라의 임신성 당뇨병의 유병률은 계속 증가할 것으로 보인다. 이는 개발 도상국을 포함한 여러 국가에서 임신성 당뇨병의 유병률이 30%이상으로 증가한 것과 유사한 추세이다[5].

임신성 당뇨병이 임산부, 태아 및 신생아의 건강에 미치는 영향은 잘 알려져 있다. 임신성 당뇨병 임산부가 정상 임산부에 비해 전자간증이 1.81배, 어깨 난산이 2.74배, 그리고 제왕절개술 비율은 1.46배 발생 위험이 커지고[6], 심리적으로도 공포 및 불안과 우울이 증가하고 스트레스 및 긴장과 유의한 연관성을 보인다[7]. 더욱이 다음 임신에서 임신성 당뇨병이 재발할 확률은 48%로 나타났고, 제2형 당뇨병의 누적 발생률은 분만 후 6주부터 28년 사이의 추적 기간에 2.6%에서 70%까지 이르면서 정상 임신 여성보다 7배의 위험률을 보인다[2]. 한편, 임신성 당뇨병을 가진 임산부에게서 태어난 태아 및 신생아는 거구증, 조산, 분만 시 외상, 신생아 저혈당증, 호흡곤란 및 입원의 위험이 증가한다[8,9]. 이러한 문제들로 인해 임신성 당뇨병의 관리와 중재가 더욱 필요하다.

임신성 당뇨병의 관리 원칙은 식이, 운동, 생활 방식의 변화를 통해 정상 수준의 혈당을 유지하고 건강한 생활 방식을 유지함으로써 임신 결과를 개선하는 것으로, 이를 위해 의학, 보건 영양 등 지금까지 다양한 중재연구들이 임신성 당뇨병 임산부에게 시도되었고 신체적, 심리적 건강지표에서 효과를 검증하였다[10]. 그러나 간호학 연구는 신체적 지표뿐 아니라 우울, 자기효능감, 모성 정체성 등의 심리 사회적 지표와 임산부 중심의 자가 관리 및 지지체계 강화 등 환경적 요인까지 고려한 복합적 중재로 그 효과가 증명된 연구들이 있었다[11]. 체계적 고찰을 통해 기존 중재들의 효과를 평가함으로써 임신성 당뇨병 임산부를 위한 유용한 중재의 핵심을 선별하고 그에 기반한 간호교육 및 중재프로그램을 임상에서 적용할 수 있기 때문에 본 연구를 실시하였다.

### 연구 목적

본 연구의 목적은 임신성 당뇨병을 진단받은 임산부를 대상으로 한 간호중재 선행 연구들의 연구방법, 중재방법 및 그 효과를 고찰함으로써, 산과 임상영역에서 활용할 수 있는 간호중재프로그램의 근거를 제시하고자 시행되었다.

## Methods

Ethics statement: This study was excluded from approval by the Institutional Review Board as a study using literature from previously published studies.

### 연구 설계

본 연구는 임신성 당뇨병을 진단받은 임산부를 대상으로 한 간호중재프로그램의 효과에 관한 선행 연구들을 분석하고, 임신성 당뇨병 간호중재연구의 효과를 파악하여 보고하기 위한 체계적 문헌고찰로, PRISMA (Preferred Reporting Items for Systematic Review and Meta-analyses)의 체계적 문헌고찰 보고지침에 따라 수행하였다.

### 문헌 선정 기준

문헌 선정을 위한 구체적인 질문은 PICO-SD (Participants, Intervention, Comparisons, Outcomes, Timing, Study Design)를 포함하였고 구체적 기준은 다음과 같다.

• 대상자(participants): 임신성 당뇨병을 진단받고 다른 임신성 합병증이 없는 임산부

• 중재 (intervention): 임신성 당뇨병을 진단받은 임산부에게 적용된 간호중재프로그램

• 비교군(comparisons): 임신성 당뇨병 관련된 간호중재프로그램을 제공받지 않은 임산부

• 결과(outcomes): 임신성 당뇨병의 결과변수는 임산부, 태아, 신생아 측면에서 제시된다. 본 연구에서는 신체적 결과 지표(체중, 임신 기간 중 체중변화, 체질량지수 등), 신진대사성 결과 지표(혈당, 당화 혈색소), 출산 결과 지표(제왕절개술, 난산, 유도분만), 심리적 안녕 결과 지표(우울, 불안, 스트레스, 자기효능감, 삶의 질, 모아 애착), 태아, 신생아 결과 지표(과체중아, 신생아 저혈당증 등)의 5가지 측면의 변수들을 모두 고려하고자 하였다.

• 연구유형(study designs): 무작위 대조군 실험연구(randomized controlled trial, RCT)만을 포함하였다.

### 문헌검색 전략

문헌검색과 분석은 2019년 12월 1일부터 2020년 2월 29일까지 이루어졌다. 인터넷을 활용한 국외 데이터베이스는 MEDLINE, Embase, Cochrane CENTRAL, CINAHL, PsycINFO이고, 국내 데이터베이스는 Koreamed, Kmbase, KISS (Korean Studies Information Service System), NDSL (National Digital Science Library), KSITI (Korea Institute of Science and Technology Information)을 통해 출판 및 완성된 학술지를 검색하였으며, 출판연도는 International Association of the Diabetes and Pregnancy study Group과 Carpenter-Coustan 두 가지 진단 기준을 다 적용할 수 있는 2000년 이후의 간호중재프로그램으로 제한하였다.

검색어는 국내의 경우 ‘임신성 당뇨병’ OR ‘임신성 당뇨’ OR ‘임신 중 고혈당’ OR ‘임신성 고혈당’ OR ‘임신 중 당뇨’ AND ‘식이’ OR ‘영양’ OR ‘식사’ OR ‘운동’ OR ‘생활습관’ OR ‘자기관리’ OR ‘자가관리’ OR ‘심리’ OR ‘교육’ OR ‘중재’ OR ‘프로그램’ OR ‘간호’ 등으로 하였다. 국외의 경우 ‘Gestational diabetes mellitus’ OR ‘pregnancy diabetes’ OR ‘Hyperglycemia in pregnancy’ OR ‘gestational hyperglycemia’ OR ‘diabetes in pregnancy’ AND ‘self-management’ OR ‘programs’ OR ‘educational programs’ OR ‘life style’ OR ‘life-style intervention’ (diet OR exercise OR physical activity OR weight) OR ‘treatments’ OR ‘psychosocial management’ OR ‘spirituality’ OR ‘nursing’ 등을 병합하여 실시하였다.

검색된 문헌 중 한국어나 영어로 출판되지 않은 연구, 출판되지 않은 학위 논문, 종설, 질적연구(사례연구, 면담에 의한 서술연구), 메타분석, 비 무작위배정 비교 임상시험연구(non-RCT), 비 무작위 연구(코호트 연구, 단면연구, 전후 비교연구), 간호사가 중재하지 않은 경우는 분석대상에서 제외하였다.

두 명의 연구자가 국내외 데이터베이스에서 제시된 검색어를 통해 문헌을 독립적으로 검색하고 문서관리 프로그램인 EndNote X9 프로그램(Clarivate Analytics, Philadelphia, PA, USA)을 이용하여 중복된 문헌을 삭제하였다. 이후 제목과 초록을 단계적으로 검토하여 선정 및 배제 기준을 적용하였으며, 초록만으로 문헌 선택 여부 결정이 어려운 경우는 논문의 전문을 일일이 찾아 검토하여 선정기준에 일치되는지 확인하였다. 그리고 의견의 불일치가 있는 경우와 최종 논문의 전문 검토 단계에서는 연구자들이 함께 충분한 논의를 통해 결과를 수렴하여 최종 논문을 결정하였다.

### 개별 연구의 비뚤림 위험 평가

두 명의 연구자가 선택된 문헌의 비뚤림 위험 평가를 위해 RCT 평가 도구인 Revised Cochrane Risk-of-Bios tool (RoB2) [12]을 사용하여 독립적으로 평가하였다. RoB2는 기존에 사용되고 있던 ROB를 2019년에 수정 보완한 도구로, 개별 무작위 평가를 위해 무작위 과정에서 발생하는 치우침, 의도된 개입으로 인한 치우침, 누락된 결과로 인한 치우침, 결과 측정의 치우침, 결과 보고 선택의 치우침의 5가지 영역으로 구성되어 있다. 각 영역마다 총 22개의 신호 전달 질문이 포함되어 있으며 그 질문에 대한 응답은 ‘그렇다(yes)’, ‘아마도 그럴 것이다(probably yes)’, ‘아마도 아닐 것이다(probably no)’, ‘아니다(no)’, ‘정보 없음(no information)’으로 평가하게 된다. 각각의 응답을 근거로 하여 비뚤림 편향을 판단하는 알고리즘에 따라 ‘편향 위험이 낮음(low risk)’, ‘일부 우려 사항(some concerns)’, ‘편향의 위험성이 높음(high risk)’으로 최종 위험 판단을 하게 된다. 연구자 간의 비뚤림 평가에 대한 의견 불일치는 토론을 통해 해결하였다.

### 자료 추출과 분석방법

체계적 문헌고찰을 위한 자료 추출 양식은 연구자들이 합의하여 항목을 결정하였으며, 이 양식에는 연구 정보(저자, 출판연도, 연구국가), 연구 대상(연령, 임신 주수, 총 참여자수), 이론 고찰, 중재방법(중재집단 수, 중재 내용, 중재기간), 연구 결과 등을 포함하였다. 한편, 본 연구는 RCT만을 포함하였지만 연구의 이질성과 중재의 복합성으로 인해 중재 방법의 효과크기를 결정할 수 없어 메타분석을 실시하지 않았다.

## Results

### 문헌 선정

**Figure 1.**
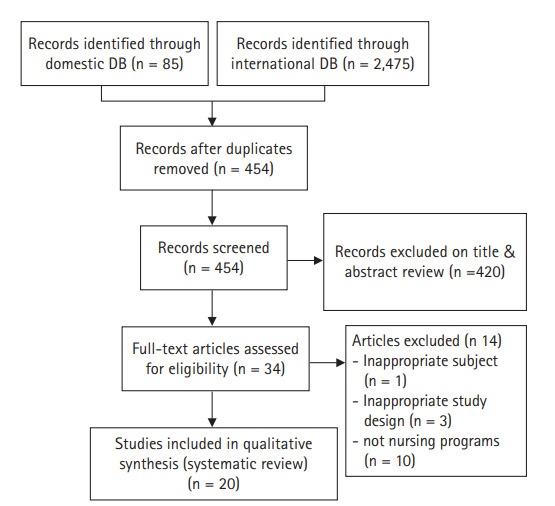
PRISMA flow chart. DB, database.

국내외 데이터베이스 검색을 통해 중복된 연구를 제외하고 일차적으로 검색된 문헌은 총 454편이었다. 문헌 선정 기준인 대상자, 중재 및 비교군, 연구유형에 따라 검색된 문헌의 제목과 초록을 검토하여 34편이 선택되었다. 선정된 34편의 문헌은 전문을 확인하였고, 그 중에서 대상자가 부적합한 문헌 1편, 연구 설계가 선정기준에 부적합한 문헌 3편, 간호중재프로그램이 아닌 문헌 10편 등 총 14편을 제외하고 20편의 논문이 최종 선정되었다[13-32] (Figure 1). 구체적으로 임신성 당뇨병 전증(prediabetic)의 임산부들을 대상으로 한 연구, 비동등성 대조군 유사 실험연구들, 의학적 중재프로그램 연구들이 제외되었다.

### 선정된 문헌의 특성

최종 체계적 문헌고찰에 포함된 연구는 총 20편으로 분석 대상 논문의 특성은 다음과 같다(Table 1). 연구 발표연도는 2002년부터 2019년도까지 분포하였으나, 2015년 이후 발표된 연구가 15편(75%)이었고 2005년까지는 1편의 연구만 보고되었다. 연구가 진행된 나라들은 이란 6편(30%), 미국 5편(25%)으로 다수였고, 오스트레일리아와 중국이 각각 2편(10%) 등이었다. 20편의 연구들 중 이론적 고찰이 이루어진 연구는 3편(15%)이었다. 연구 장소는 20편(100%) 모두 병원이며, 중재가 개인별로 적용된 연구가 19편, 그룹별인 경우는 1편이었다. 대상자의 수는 38명부터 233명까지 다양하였고 대상자의 나이는 30대 이상을 대상으로 한 연구가 15편(75%)인 반면, 각 군별로 평균 나이가 다른 경우가 1편(5%), 제시되어 있지 않은 연구가 1편(5%)이었다. 대상자의 임신 주수는 12주부터 분만 시(intrapartum)까지 다양하게 나타났다.

### 문헌의 비뚤림 위험 평가

본 연구에 포함된 20편의 연구를 RoB2 도구를 이용하여 비뚤림 위험을 평가하였고, Cochrane 그룹에서 제공하는 Review Manager ver. 5.4 (Cochrane, London, UK)를 이용하여 분석하였다. 그 결과 첫 번째 영역인 무작위배정 과정(randomization process) 영역에서 무작위배정 과정을 상세히 기술하고, 중재군과 대조군의 기준선의 차이가 없어 ‘low risk’로 판단된 연구가 14편(70%), 무작위 과정에 대해 상세한 설명이 없지만 대상자에 대한 기준 또는 배정 비율은 같아 ‘some concerns’로 판단된 연구가 6편(30%) [14,16,25,27,29,31]이었다.

의도된 중재(intended interventions), 누락된 결과(missing outcome data), 결과 보고(reported result) 영역에서는 모두 비뚤림의 위험이 낮다고 평가되었다.

**Figure 2.**
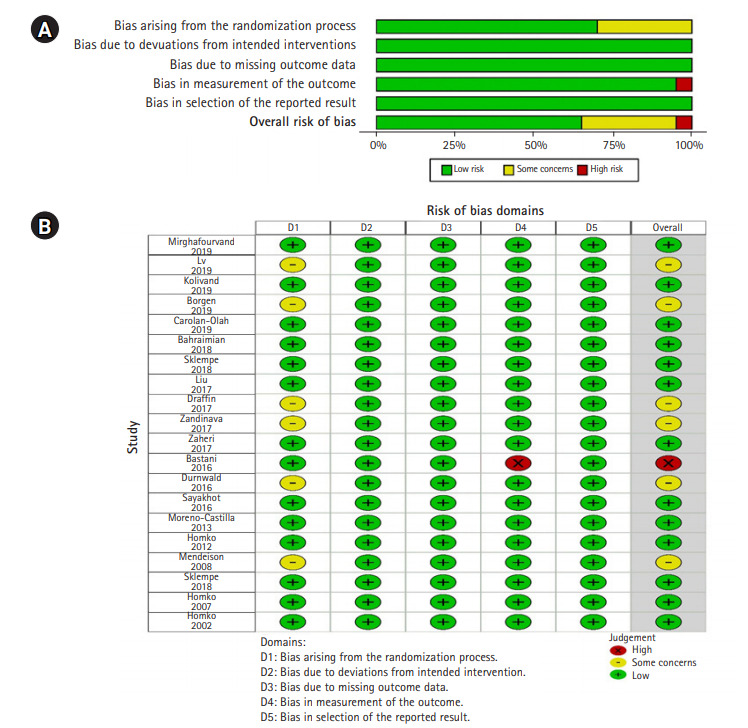
Risk of bias graph. (A) Risk of bias summary. (B) Risk of bias for selected studies.

결과 측정(measurement of the outcome) 영역에서는 결과 측정 방법이 부적절하여 연구 결과에 영향을 미칠 영향이 크다고 판단하여 ‘high risk’로 판단한 연구가 1편(5%)이고[23], 나머지 19편(95%)은 결과 측정 방법이 적절하여 ‘low risk’로 판단되었다(Figure 2A).

### 체계적 문헌고찰 결과

간호중재프로그램의 종류로는 단일 중재로 식이 2편, 운동 2편, 스트레스 관리 1편, 혈당 모니터링 3편과 보완요법 2편이고, 통합 중재프로그램은 10편이었다. 중재 기간은 짧게는 3일에서 길게는 분만 시였고, 중재의 효과측정 시기 또한 중재 직후, 2-4주 후, 출산 후 등 다양하였다. 측정된 효과변수는 혈당을 포함하여 임산부, 태아, 신생아 결과 지표와 심리적 안녕 지표로 다양하게 나타났다(Table 2).

#### 통합 중재프로그램

영양, 운동, 생활 습관과 혈당 관리 중 2가지 이상의 중재를 통합한 프로그램의 효과를 평가한 연구들로 모두 10편이었다. 혈당 관련 신진대사성 결과지표와 스트레스, 자기효능감, 자기 관리 관련 심리적 안녕 결과지표가 가장 두드러진 효과로 나타났다.

##### 1) 직접적인 대면 교육을 통한 중재

중국 임산부 120명이 대상인 연구에서 임신성 당뇨병 관련 지식, 운동, 혈당 모니터링 및 정신건강 교육 등을 제공받은 중재군의 혈당 조절, 생활습관 변화, 질환 관련 지식 인식률, 공복 및 식후 2시간 혈당이 향상되었고, 조산, 산후 출혈, 양수과다증, 태아 곤란, 요로감염 발생률 및 신생아 관련 합병증도 통계적으로 낮게 나타났다[23]. 멕시코계 미국 임산부를 대상으로 한 연구는 임신성 당뇨병의 유형 및 위험요인, 영양, 활동 및 의학적 관리, 식품군과 측정 관련 영양요법에 대해 1시간 교육을 제공하고 3주 후 측정한 중재군의 건강 책임, 신체 활동, 영양, 영적 성장, 대인관계 및 스트레스 관리를 측정하는 항목이 포함된 건강 증진 생활습관 프로파일 점수에서 유의한 차이가 있었다[30]. 한편, 이란 임산부들을 대상으로 4주 동안 총 4회의 교육을 실시한 결과, 자기관리 행동과 혈당 수치에서 효과를 보였다[21].

##### 2) 디지털 영상자료(digital video disc, DVD), 웹 또는 모바일 기반 교육 프로그램 중재

영국 임산부들에게 46분 분량의 DVD 프로그램과 대면 교육을 함께 제공한 연구에서는 중재군에서 아침 식후 1시간 혈당이 상당히 낮았고, 출산력을 조정한 제왕절개술 비율에도 통계적으로 유의한 차이가 있었다[24]. 오스트레일리아 임산부 120명을 대상으로 12주간 온라인 교육 프로그램을 실시한 결과 중재군에서 체중 및 체질량지수, 모성의 혈압, 혈당 및 경구 당부하검사 수치에서 효과가 나타났다[16]. 그러나 오스트레일리아 임산부들에게 대면 교육과 웹 기반 프로그램을 같이 적용한 중재[25]와 노르웨이 임산부들에게 모바일 프로그램을 적용하고 혈당 수치를 저장 및 전송하게 한 중재[17]는 효과가 없었다.

##### 3) 자가 관리 행동의 강화를 위한 중재

이란 임산부 151명을 대상으로 한 연구에서 자가 관리 패키지 및 3회의 대면 교육을 실시한 결과, 중재군에서 식후 2시간 혈당, 신생아 아프가(Apgar) 점수와 자기효능감, 신생아 입원율이 통계적으로 유의한 차이를 보였다[15]. 앞서 제시한 연구와 다른 이란 임산부들에게 매주 1시간씩 4번의 교육을 적용하였더니 제왕절개술, 거구증에서 효과가 나타났다[13]. 반면, 미국에서 101명의 임산부들을 대상으로 최소 두 번의 영양 상담, 임신과 산후 관리에 관한 책자 제공 및 매주 20–30분간의 전화 상담으로 구성한 집중 행동교육 프로그램은 효과가 나타나지 않았다[26].

#### 단일 중재 프로그램

혈당 모니터링 연구 3편, 영양 2편, 운동 2편, 심리 치료 요법 1편, 보완요법 2편으로 총 10편이었다. 혈당 관련 신진대사성 결과지표와 스트레스, 자기효능감, 자기 관리 관련 심리적 안녕 결과지표가 가장 두드러진 효과로 나타났다.

##### 1) 혈당 모니터링

미국 저소득층 임산부들에게 인터넷을 통한 혈당 모니터링을 실시하였더니 자기효능감이 증진되었다[31]. 한편, 미국 임산부 58명을 대상으로 실시한 혈당 자가 모니터링 중재[32]와 미국 임산부 80명을 대상으로 인터넷과 전화를 이용하여 실시한 혈당 감시 모니터링 중재[29]는 효과 평가에서 통계적으로 유의하지 않았다.

##### 2) 영양

중국 임산부들을 대상으로 저당부하 식이를 적용하였더니 공복혈당, 식후 2시간 혈당, 조산, 거구증, 자간증 및 임신성 고혈압, 태아 곤란 관련 지표에서 효과가 나타났다[14]. 그러나 스페인 임산부 150명을 대상으로 저탄수화물 식이를 실시한 결과, 유의한 효과가 없었다[28].

##### 3) 운동

크로아티아 임산부 38명을 대상으로 에어로빅과 저항운동을 결합한 운동 프로그램을 50–55분 동안 일주일에 2번씩 임신 기간 내내 실시하도록 하였더니 중재군에서 식후 2시간 혈당과 임신 중 합병증에서 유의한 효과가 나타났다[18]. 또한 터키 임산부들에게 복식호흡을 매일 아침 5분 동안 하도록 한 결과, 불안, 우울, 스트레스가 감소하고 모아 애착은 높아진 것으로 나타났다[19].

##### 4) 심리 치료 요법

80명의 이란 임산부들을 대상으로 심리 치료 요법 중 하나인 인지행동요법을 1회당 2시간씩 6회의 교육을 3주간 적용한 효과를 측정하였더니 사후 스트레스 점수와 공복혈당이 유의하게 감소하였다[22].

##### 5) 보완요법

이란 임산부들에게 500 mg 생강 캡슐을 1일 2회씩 8주간 복용하도록 한 결과, 식후 2시간 혈당, 인슐린 투여량 및 산부인과 방문 빈도가 줄었다[20]. 한편, 다른 이란 연구에서 병원에 입원 중인 60명의 임산부들을 대상으로 3일 동안 30분씩 중재군에게 지압을 적용한 효과를 측정하였더니, 임산부의 불안도와 그 심각도가 유의하게 감소하였다[27].

## Discussion

본 연구는 임신성 당뇨병 임산부를 위해 개발, 적용된 간호중재 연구 중 RCT를 대상으로 연구방법, 중재내용 및 결과를 종합하여 고찰하기 위해 시도하였다. 검색체계에 따라 추출된 문헌 20편을 분석한 결과, 실험중재가 제공된 장소는 모두 병원이었고, 연구 대상자의 수는 다양하였다. 관리를 위해 가장 많이 시도된 중재는 통합 중재로 영양, 운동, 생활습관과 혈당 관리 중 2가지 이상의 중재를 조합한 것이었고, 단일 중재로는 혈당 모니터링, 영양, 운동, 심리 및 보완요법 등이 적용되었다. 중재의 결과로서 측정한 종속변수 역시 다양하게 나타났는데 혈당 수치인 공복혈당, 식후 2시간 혈당, 경구 포도당부하 검사가 가장 많이 측정되었다. 한편, 본 연구의 간호중재프로그램에서 신진대사성 결과 지표와 심리적 안녕 결과 지표가 가장 효과적인 것으로 나타났다.

임신성 당뇨병 임산부들을 위한 통합 중재요법은 임신성 당뇨병에 대한 지식, 영양, 운동, 혈당 모니터링 및 정신건강을 조합한 프로그램이 다수였고, 이러한 프로그램 중재 전달 형태는 2000년 초에는 대면 교육 프로그램이었으나[21,23,30] 이후 DVD [24], 웹[16,25], 또는 모바일 기반[17]의 교육으로 변화를 도모하여 산모, 태아 및 신생아 결과 지표에서 효과를 나타내고 있었다. 이는 11개의 임신성 당뇨병 모바일 앱의 효과를 고찰한 연구에서 혈당 수준을 엄격하게 조절하고 임신성 당뇨병 관련 위험을 효과적으로 관리하고 예방할 수 있음이 입증된 맥락과 일치한다[33]. 이러한 결과를 토대로, 사회적 환경이나 예측하지 못한 상황이 발생한 경우 직접 대면 교육이 불가능할 때, 웹 또는 모바일 기반의 교육 및 행동 프로그램의 유용성이 높아지고 있어 미래의 간호대상자들에게 부합하고 활용가능성이 높은 중재를 개발, 시도할 필요가 있다.

통합 중재프로그램은 혈당 관련 신진대사성 결과지표와 스트레스, 자아정체성, 자기 관리 관련한 심리적 안녕 결과 지표에서 가장 효과적이었다. 이는 통합 중재프로그램을 고찰한 연구에서 신생아 과체중[34,35], 거구증[34,36], 혈당 감소[11,37], 우울[11,36] 등의 효과가 나타난 결과 지표와 비교했을 때 어느 정도 일치하는 부분이 있었다. 그러나 태아, 신생아 결과 지표에서 효과를 나타내지 못한 점에 주목해서 추후 임신성 당뇨병 관련 간호중재프로그램 구성 시 반영해야 할 것이다.

단일 중재프로그램 중 다수를 차지한 혈당 모니터링 중재는 중재 환경, 중재 빈도, 그리고 모니터링 방법이 연구마다 서로 달랐고 결과적으로 산모, 태아 및 신생아 관련 결과 지표에서의 효과가 일관되게 나타나지 않았다[29,31,32]. 이는 11개의 RCT를 통해 혈당 모니터링 중재의 효과를 고찰한 결과, 전자간증 또는 임신성 고혈압, 제왕절개술, 유도분만, 거대 중량아, 임산부의 사망 또는 중증 이환율 또는 신생아 저혈당증 또는 사산율에서 명확한 차이가 나타나지 않았다는 결과와 일관된 결과이다[38]. 이러한 결과를 토대로, 혈당 모니터링 중재프로그램 구성 시 대상자 수, 모니터링 방법, 측정 시기, 측정 빈도, 측정 결과에 따른 피드백 변화를 주는 반복적인 연구가 요구된다.

식이요법 중재의 효과를 종합한 결과, 저탄수화물 식이 중재가 임신성 당뇨병 임산부의 중요한 치료 전략이나 본 연구에서 가장 효과적인 식단은 저당부하 식이로 나타났다. 이는 식이요법 중재프로그램 고찰 연구에서 저당부하 식이가 거구증[39], 신생아 체중 및 인슐린 사용 감소[40,41]에 의미가 있다는 결과와 일치하고 있다. 그러나 저탄수화물 식이 중재는 유의한 효과가 나타나지 않았는데[28], 이는 저탄수화물 식이 중재 시 오히려 식후 혈당이 상승하고 태아 지방이 높아졌다는 결과와 맥락을 같이한다[42]. 이러한 결과를 토대로, 보편적인 치료 전략인 저탄수화물 식이보다 저당부하 식이가 효과적인 식이 전략으로 나타났는데, 국내 임신성 당뇨병을 가진 임산부들에게 더 적합한 식이 전략을 구현하기 위한 반복 연구가 요구된다.

에어로빅과 저항운동을 결합한 프로그램은 혈당과 신생아 체중 감소에 효과적이었는데[18], 이는 임신성 당뇨병을 가진 임산부를 대상으로 실시한 운동 요법 고찰연구에서 혈당 감소[43], 인슐린 요구량 감소[43,44], 거대아, 제왕절개술 비율 감소[34] 효과와 일치하는 것으로 나타났다. 그러나 모아 애착, 불안 및 우울에 효과적인 복식호흡[19] 중재와 관련하여 임산부를 대상으로 한 연구는 본 연구에서 다룬 1편으로 반복 연구가 요구된다.

인지행동요법은 스트레스 감소뿐 아니라 혈당 감소에도 효과를 나타냈는데[22], 제2형 당뇨병 및 동반성 우울증이 있는 성인 환자를 대상으로 생활습관 상담을 통한 맞춤 인지행동 치료요법을 실시하여 당화혈색소 수치가 개선된 결과가 일치한다고 할 수 있다[45]. 그러나 인지행동 요법과 관련한 임신성 당뇨병 임산부 대상 연구는 본 연구에서 다룬 1편으로 반복 연구가 요구된다.

보완요법으로 생강 캡슐 복용은 식후 혈당 및 인슐린 요구량을 감소시켰으나[20], 이란의 임신성 당뇨병 임산부 70명을 대상으로 실시한 연구는 6주간 생강 캡슐 복용한 중재군에서 공복혈당, 공복 인슐린의 평균은 감소하였으나 식후 2시간 혈당은 유의한 차이가 없는 것으로 나타났다[46]. 이는 생강 캡슐 복용이 혈당 및 인슐린 요구량의 감소에 어느 정도 효과가 있다고 볼 수 있다. 한편, 지압은 임신성 당뇨병으로 인한 불안감을 낮추는 데 효과적인데[27], 30명의 임신성 당뇨병 임산부를 대상으로 한 이집트 연구에서 12주간 지압 실시 후 75 g 경구 당부하검사, 인슐린 저항성, 인슐린 수치 및 인슐린 치료 횟수가 상당히 감소한 것으로 나타났다[47]. 이는 지압 요법이 신체적, 심리적 결과 지표에서 효과를 나타내는 것으로 볼 수 있다. 이러한 결과를 토대로, 생강 캡슐 복용과 지압 요법과 같은 보완요법에 대한 국내 임신성 당뇨병을 가진 임산부들에 대한 효과를 측정하기 위한 반복 연구가 요구된다.

본 연구에서 비뚤림 위험 평가 결과 비뚤림의 위험이 높았던 연구는 전체의 5%로, 이로 인해 효과가 과대 추정될 가능성이 존재하므로 연구 결과 해석 시 주의해야 하며 이를 보완한 연구가 요구된다.

이상의 결과를 토대로, 임신성 당뇨병 임산부를 대상으로 한 간호중재프로그램의 기존 연구의 결과가 유의하고 대부분의 개입이 어느정 도 가치가 있음을 발견하였다.

첫째, 통합 중재 프로그램의 구성이나 제공 방식이 계속 진화하고 있으며, 신진대사성 및 심리적 안녕 지표에서 효과를 나타내고 있다.

둘째, 혈당 모니터링 요법은 중요한 치료 전략이지만 그 효과성을 입증하지 못하고 있다. 그럼에도 불구하고, 계속적으로 다양한 중재 전략이 수립되어 연구가 시도되고 있는 것은 의의가 있다.

셋째, 혈당 모니터링, 영양, 운동 등 신체적 건강지표에 미치는 중재가 물론 중요하나 다수의 연구에서 임신성 당뇨병 임산부가 겪는 심리적 어려움 또한 드러난 점을 고려할 때 불안, 우울, 스트레스, 자기효능감 및 자기 관리와 관련한 심리적 안녕 지표를 간호중재에 포함한 프로그램 또한 유용성이 높은 것으로 나타났다.

본 연구는 RCT로만 구성되었으므로 전반적으로 낮은 비뚤림 위험을 보여 연구의 질이 높은 것으로 평가된다. 또한 연구 간의 이질성에도 불구하고 임신성 당뇨병을 가진 임산부들을 위한 단일 및 통합 간호중재에서 임산부, 태아, 신생아 측면의 효과를 발견하였으며, 효율적인 간호중재프로그램을 구축하기 위한 구체적인 증거를 제시함으로써 임신 중, 산후 및 신생아 건강을 개선하는 데 실질적으로 기여할 수 있는 방법을 제공하였다는 데 의의가 있다. 그러나 고찰된 연구가 서로 다른 중재 전략을 구현하고 있고 일관되지 않은 결과 지표를 보고하고 있으므로 확실한 효과를 단정짓기 어렵고, 일반화된 중재프로그램을 제시하기에는 제한점이 있다. 고찰된 연구들이 국외 연구들로 구성되어 있어 국내 현실을 반영한 간호중재프로그램에 그 효과를 입증할 반복 연구들이 요구되고, 중재 전달 방식 또한 시대적 상황을 반영한 컨텐츠를 개발하고 효과를 검증할 것을 제언하는 바이다.

## Figures and Tables

**Table 1.** General characteristics and research methodology of the included studies (N=20)

Variable	Categories	n (%)	References
Publication year	2000–2004	1 (5.0)	[32]
	2005–2009	2 (10.0)	[30,31]
	2010–2014	2 (10.0)	[28,29]
	2015–2019	15 (75.0)	[13-27]
Country	Iran	6 (30.0)	[13,15,20-22,27]
	United States of America	5 (25.0)	[26,29-32]
	Australia	2 (10.0)	[16,25]
	China	2 (10.0)	[14,23]
	Norway, Turkey, Spain, Croatia (each)	1 (5.0)	[17-19,28]
	United Kingdom	1 (5.0)	[24]
Use of a theoretical framework	Yes	3 (15.0)	[14,17,30]
	No	17 (85.0)	[13,15,16,18-29,31,32]
Intervention setting	Hospital	20 (100)	[13-32]
Intervention unit	Individual	19 (95.0)	[13-23,25-32]
	Group	1 (5.0)	[24]
Sample size of each group	<50	10 (50.0)	[13,18-22,27,29,31,32]
	50–79	9 (45.0)	[14-16,23-26,28,30]
	≥80	1 (5.0)	[17]
Mean age of participants (year)	<30 in both the control and experimental groups	3 (15.0)	[22,27,31]
	≥30 in both the control and experimental groups	15 (75.0)	[13,15-21,23-26,28-30]
	Different range of mean age at each group	1 (5.0)	[32]
	Not presented	1 (5.0)	[14]

**Table 2.** Characteristics of selected studies (N=20)

First author, year [reference]	Country	Selection criteria	Int.	Cont.	Measurement: result (statistical significance)	
Mirghafourvand, 2019 [13]	Iran	IUP 28-30 weeks	n=46	n=46	Post-delivery:	
			Self-care training (physical activity and nutrition); 4 one-hour sessions per week.	Usual care	C/S, macrosomia (+)	
					Birth weight, height, and head circumference, preterm labor (–)	
Lv, 2019 [14]	China	Not presented	n=67	n=67	Pre - IUP 37 weeks: FBG and 2-hour PPG levels (+) premature delivery, fetal macrosomia, eclampsia, pregnancy hypertension syndrome, and fetal distress (+)	
			Nutritional nursing education based on GL (glycemic load); Duration not presented.	Usual care		
Kolivand, 2019 [15]	Iran	IUP 20-30 weeks	n=75	n=76	Pre - Post 1 month: Maternal self-efficacy, 2-hour PPG, Apgar scores, neonatal hospitalization (+)	
			Self-care guidebook, educational DVDs, educational software, logbook, and three face-to-face educational sessions; 7 weeks	Usual care	Neonatal age at delivery, delivery type (–)	
Carolan-Olah, 2019 [16]	Australia	IUP 28-32 weeks	n=52	n=58	Pre-12 weeks PP: PP weight, OGTT at 12 weeks (+); maternal blood pressure, neonatal birthweight (–)	
			An online educational program (healthy food choices, healthy habits/healthy lifestyle, emotions, family and food, and testing blood glucose levels)	Usual care		
Borgen, 2019 [17]	Norway	<IUP 33 weeks	n=112	n=121	IUP 36 weeks – Birth –PP 3 months: Proportion of emergency C/S (+)	
			The Pregnant+ app (healthy diet, being physically active and feedback on BG); Duration not presented.	Usual care		
					PP OGTT 2-hour BG level, birth weight, breast feeding practice, obstetric complications or transfer to the intensive neonatal care unit (–)	
Sklempe, 2018 [18]	Croatia	IUP 30-36 weeks	n=18	n=20	IUP 30^th^ - 33^rd^ - 36^th^ week:	
			Regular supervised exercise and daily brisk walks of at least 30 min (20 min aerobic, 20-25 min resistance, pelvic floor, stretching, 10 min of relaxation); 2weeks (50-55 min sessions)	Usual care	PPG levels at end of pregnancy, neonatal BMI (+),	
Fiskin, 2018 [19]	Turkey	IUP 24-28 weeks	n=30	n=30	Pre - Day 5 – Day 30:	
			Diaphragmatic breathing exercise (5 minutes every morning; questionnaires twice a month); 30 days	Usual care	Psychological state (depression, anxiety, stress), maternal-fetal attachment (+)	
						
Bahramian, 2018 [20]	Iran	IUP 24-28 weeks	n=38	n=38	Every 2 weeks:	
			500 mg ginger capsules twice a day; 8 weeks	Placebo capsules twice a day	Mean 2 hour PPG, insulin dose, and frequency of obstetrical visits (+)	
					FBS, hemoglobin A_1_C (–)	
Zandinava, 2017 [21]	Iran	IUP 28-30 weeks	n=46	n=46	Pre – Post 4 weeks:	
			Self-care educational with educational booklet; 4 weeks	Usual care	Self-care behaviors, BG at 1 hour and 2 hours after GTT (+); FBS, quality of life (–)	
Zaheri, 2017 [22]	Iran	IUP 24-32 weeks	n=40	n=40	Pre – Post 2 weeks:	
			Cognitive-behavioral stress management; six 2-hour sessions; 3 weeks	Usual care	Stress (+)	
					FBS (–)	
Liu, 2017 [23]	China	IUP 24-37 weeks	n=60	n=60	Every month: BG control, lifestyle change and knowledge on GDM (+); incidences of premature birth, PP hemorrhage, hydramnios, fetal distress, and urinary infection (+); Neonatal incidence of hyperbilirubinemia, severe asphyxia, hypoglycemia and pneumonia (+)	
			Health education (mental health, exercise; medication guidance; BG monitoring; obstetric education; weight control): Duration not presented.	Usual care		
Draffin, 2017 [24]	United Kingdom	Not presented	n=77	n=73	Pre – 2 weeks later – at PP 6-8 weeks:	
			Patient-centered educational DVD (living with GDM, SMBG, administering insulin, calculating BMI post-pregnancy, healthy eating); 46 min	Usual care	Maternal anxiety at 2 weeks, 1-hour PPG, pregnancy specific stress, emotional adjustment to GDM, self-efficacy, knowledge of GDM, risk perception for developing diabetes (–)	
Sayakhot, 2016 [25]	Australia	Not presented	n=56	n=60	Post:	
			Standard GDM education with online touch screen/computer program (healthy food choices, habits/ lifestyle, emotions, family and food, and SMBG)	Usual care	Knowledge of GDM (–), knowledge of food choice and exercise during pregnancy (–), knowledge of GDM management (–), association of education levels and knowledge of GDM (–)	
Durnwald, 2016 [26]	United States	<IUP 33 weeks	n=49	n=52	At PP 6-12 weeks:	
			Intensive behavior education program (at least two nutrition counseling sessions in pregnancy and PP; focused on healthy lifestyle choices, motivational messaging, exercise and nutritional facts); 20–30 min telephone calls each week; Duration not presented.	Usual care	FBS and 2-hour PPG (–)	
Bastani, 2016 [27]	Iran	IUP 20-42 weeks	n=30	n=30	At 3 days:	
			Nurse-provided acupressure at the true point ; 30 min interval for three days	Nurse-provided acupressure at sham (false) point	Anxiety (+)	
Moreno-Castilla, 2013 [28]	Spain	≤IUP 35 weeks	n=75	n=75	At each follow up visit – after delivery:	
			Low-carbohydrate diet (40% of total diet energy content as carbohydrate and 40% fat intake; 3 principal meals and 3 snacks); Duration not presented.	Control diet (55% as carbohydrate and 25% fat; 3 principal meals, and 3 snacks)	Differences in daily carbohydrate consumption (+), rate of requiring insulin, maternal weight gain, ketonuria, maternal hypertension, C/S, small for gestational age, large for gestational age, macrosomia, newborn hypoglycemia (–)	
Homko, 2012 [29]	United States	<IUP 33 weeks	n=40	n=40	Every 2 weeks until IUP 36 weeks – then weekly:	
			Telemedicine care (to transmit BG levels, fetal movement, insulin doses and episodes of hypoglycemia; via phone/internet at least weekly): Until birth	Usual care	Maternal BG, neonatal birth weight (–)	
Mendelson, 2008 [30]	United States	IUP 12-32 weeks	n=49	n=51	At 3 weeks – delivery admission day:	
			A Parish Nurse-led discussion regarding medical recommendations for control of GDM; 1 hour	Usual care	Health Promoting Lifestyle Profile II scores (+)	
					Glycemic control, macrosomia, or days of maternal or neonatal hospitalization (–)	
Homko, 2007 [31]	United States	≤IUP 33 weeks	n=32	n=25	Every 2 weeks until IUP 36 weeks – then weekly:	
			Telemedicine care (website was established for BG documentation and communication with health care team): Until birth	Usual care	Self-efficacy (+): FBS or PPG (although more women in the Int. group received insulin therapy), Pregnancy and neonatal outcomes (–)	
Homko, 2002 [32]	United States	≤IUP 33 weeks	n=31	n=27	Pre – at IUP 37 weeks: The Diabetes Empowerment Scale (-)	
			SMBG education using a reflectance meter with memory (One Touch Profile) and SMBG four times a day (FBS and 1 hour PPG); Total of four times a week: Duration not presented.	BG levels measured (FBS and 1 hour PPG) at each prenatal visit or more frequently if clinically indicated	At each visit: Dietary compliance, birth weight, gestational age at delivery, Apgar, neonatal complications, rates of macrosomia, C/S, birth trauma (–)	

BG: Blood glucose; BMI: body mass index; Con: control; C/S: cesarean section; DVD: digital video disc; FBS: fasting blood sugar; GDM: gestational diabetes mellitus; GTT, glucose tolerance test; Int: intervention; IUP: intrauterine pregnancy; min: minutes; OGTT: oral glucose tolerance test; PP: postpartum; PPG: postprandial glucose; SMBG: self-monitoring of blood glucose.
